# Supplementary material for: Synthesis and Characterization of Lanthanide Metal Ion Complexes of New Polydentate Hydrazone Schiff Base Ligand
Source: Molecules. 2022 Dec 1;27(23):8390. doi: 10.3390/molecules27238390 (PMC9736465; doi:10.3390/molecules27238390)
Supplement: Supplementary file 1 [file molecules-27-08390-s001.zip › molecules-2020152-supplementary.pdf]

Supplementary materials

Synthesis and Characterization of Lanthanide Metal Ion Complexes of New Polydentate Hydrazone Schiff Base Ligand

Izabela Pospieszna-Markiewicz, Marta A. Fik-Jaskółka, Zbigniew Hnatejko, Violetta Patroniak and Maciej Kubicki *

Faculty of Chemistry, Adam Mickiewicz University, Uniwersytetu Poznańskiego 8, 61-614 Poznań, Poland
* Correspondence: mkubicki@amu.edu.pl

Content

1. Scheme S1. General structures of the resulting complexes (group A and B).

2. Analytical data for ligand. Figure S1. ^{1}H NMR spectrum of ligand in DMSO-d_6.

3. Fourier map Figure S2.

4. X-ray crystallography Table S1.

5. Thermal analysis of the complexes. Figure S3. Thermogravimetric analysis (TGA) curves of representative of complexes (a) 2, (b) 5, (c) 6.

6. Luminescence properties.

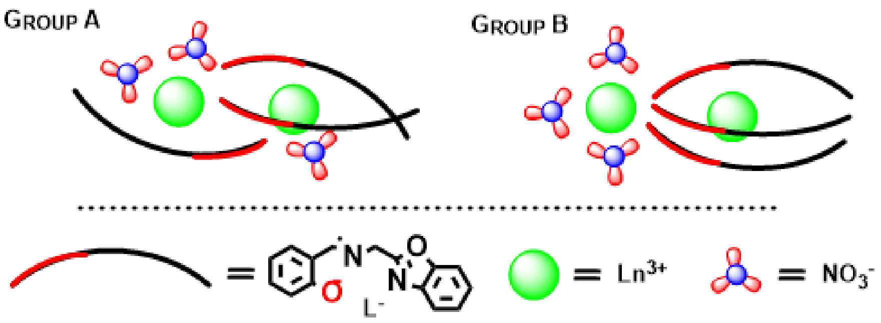

Scheme 1. General structures of the resulting complexes (group **A** and **B**).

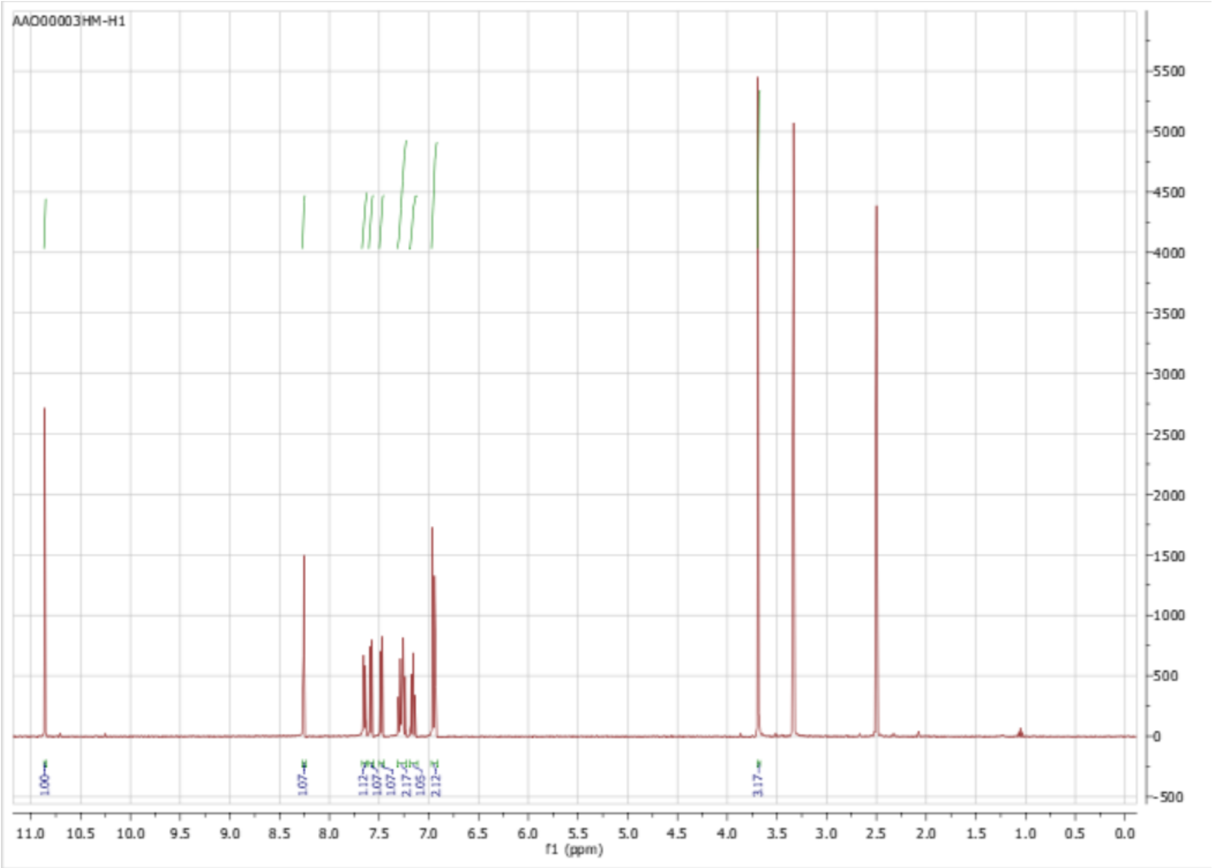

Figure S1. ^{1}H NMR spectrum of ligand in DMSO-d6.

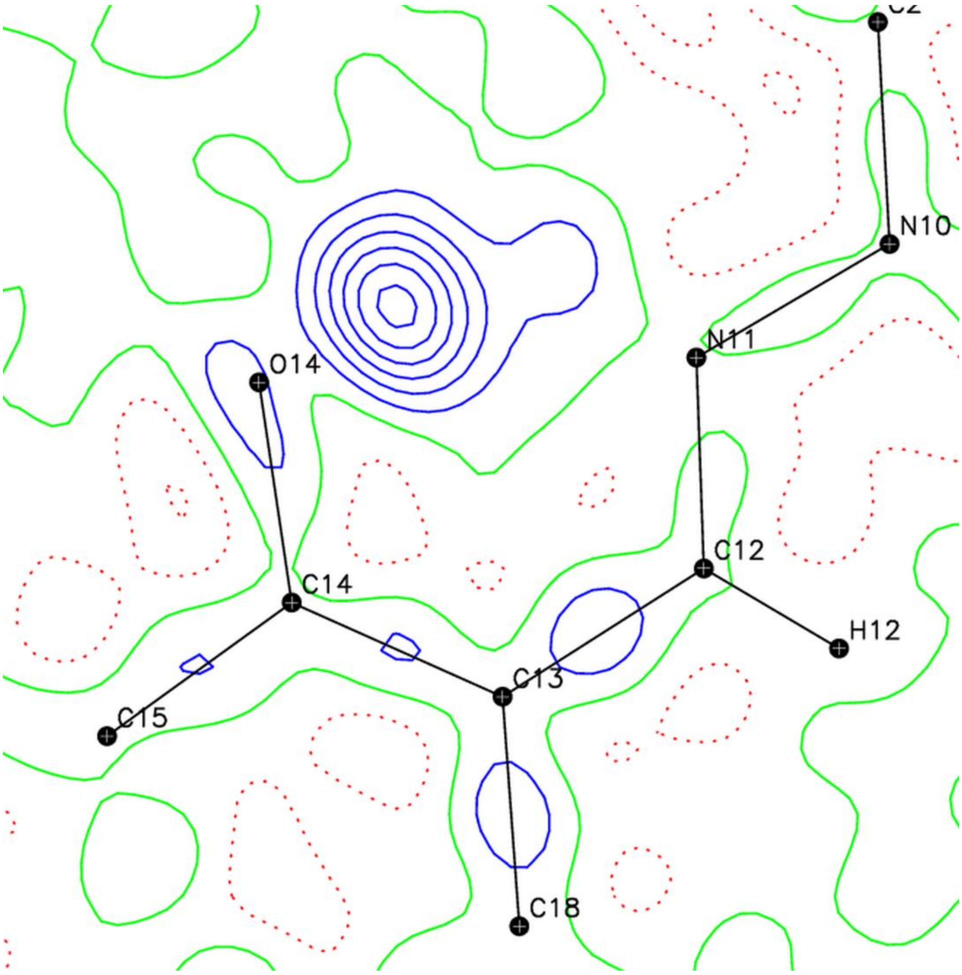

Figure S2. The difference Fourier map for the structure HL without the OH or NH hydrogen atom; the position of this hydrogen next to oxygen is clearly seen.

Table S1. Crystal data, data collection and structure refinement.

Compound	HL	1 (Sm)	2 (Eu)	3a (Tb)
Formula	$C_{15}H_{13}N_3O_2$	$C_{45}H_{36}Sm_2N_{12}O_{15}\cdot C_2H_3N$	$C_{45}H_{36}Eu_2N_{12}O_{15}\cdot C_2H_3N\cdot C_6H_{14}O$	$C_{45}H_{36}N_{12}O_{15}Tb_2\cdot C_7H_8\cdot C_2H_3N\cdot CH_4O$
Formula weight	267.28 /c	1326.61	1432.03 /c	1467.92
Crystal system	orthorhombic	monoclinic	triclinic	triclinic
Space group	Pbcn	$P2_1/c$	P-1	P-1
a(Å)	22.6342(4)	11.5200(5)	14.2703(4)	11.5819(7)
b(Å)	8.59955(12)	14.8132(5)	14.2792(4)	12.9463(7)
c(Å)	12.8361(2)	29.8965(9)	16.8854(4)	19.4420(7)
α(°)	90	90	87.661(2)	74.414(4)
β(º)	90	91.509(3)	65.209(2)	87.335(4)
γ(°)	90	90	65.232(3)	86.059(5)
V(Å^3)	2498.47(7)	5100.0(3)	2798.08(15)	2800.2(3)
Z	8	4	2	2
D_x(g cm^{-3})	1.421	1.728	1.700	1.741
F(000)	1120	2624	1432	1460
μ(mm^{-1})	0.795	2.361	2.303	2.588
Reflections: collected	5790	20471	46097	32225

unique (R_{int})	2503 (0.0173)	9788 (0.0399)	12597 (0.0273)	11795 (0.0494)
with I>2σ(I)	2277	7629	11266	9539
R(F) [I>2σ(I)]	0.0387	0.0518	0.0198	0.0436
wR(F^2) [I>2σ(I)]	0.1003	0.1218	0.0434	0.1017
R(F) [all data]	0.0425	0.0716	0.0248	0.0581
wR(F^2) [all data]	0.1042	0.1357	0.0456	0.1126
Goodness of fit	1.023	1.072	1.060	1.036
max/min Δ (e·Å^{-3})	0.19/-0.31	2.82/-2.02	0.64/-0.61	1.89/-1.66
CCDC number	1542869	2062743	2062744	1542870

**Compound**	**3b (Tb)**	**4 (Dy)**	**5 (Ho)**
Formula	$C_{45}H_{36}N_{12}O_{15}Tb_2\cdot C_2H_3N\cdot C_6H_{16}N^+\cdot NO_3^-$	$C_{45}H_{36}Dy_2N_{12}O_{15}\cdot C_2H_3N$	$C_{45}H_{36}Ho_2N_{12}O_{15}\cdot 3(C_2H_3N)\cdot CH_4O$
Formula weight	1507.96	1350.91	1469.92
Crystal system	cubic	monoclinic	monoclinic
Space group	$P2_13$	$P2_1/c$	$P2_1/n$
a(Å)	17.90482(15)	11.4753(3)	13.4899(4)
b(Å)	17.90482(15)	14.7637(4)	21.7608(8)
c(Å)	17.90482(15)	29.7478(8)	20.2951(9)
α(°)	90	90	90
β(º)	90	91.904(2)	108.887(4)
γ(°)	90	90	90
V(Å^3)	5739.97(14)	5037.0(2)	5636.9(4)
Z	4	4	4
D_x(g cm^{-3})	1.745	1.781	1.732
F(000)	3008	2656	2912
μ(mm^{-1})	2.531	3.026	2.879
Reflections:			
collected	20848	19321	27295
unique (R_{int})	4349 (0.0277)	9636 (0.0250)	10885 (0.0267)
with I>2σ(I)	4189	8460	9573
R(F) [I>2σ(I)]	0.0154	0.0256	0.0340
wR(F^2) [I>2σ(I)]	0.0311	0.0538	0.0780
R(F) [all data]	0.0174	0.0321	0.0417
wR(F^2) [all data]	0.0317	0.0563	0.0809
Goodness of fit	1.024	1.028	1.071
max/min Δ (e·Å^{-3})	0.23/-0.28	0.74/-1.18	1.35/-1.17
CCDC number	2062745	2062746	1542871

**Compound**	**6 (Er)**	**7 (Tm)**	**8 (Yb)**
Formula	$C_{45}H_{36}N_{12}O_{15}Er_2\cdot C_2H_3N$	$C_{45}H_{36}N_{12}O_{15}Tm_2\cdot 4(C_2H_3N)$	$C_{45}H_{36}N_{12}O_{15}Yb_2\cdot 4(C_2H_3N)$
Formula weight	1359.42	1482.90	1495.15
Crystal system	monoclinic	monoclinic	monoclinic
Space group	$P2_1/c$	$P2_1/c$	$P2_1/c$
a(Å)	11.51739(10)	13.6208(5)	13.60755(17)
b(Å)	14.79757(11)	21.8908(8)	21.86269(19)
c(Å)	29.90397(19)	20.4587(6)	20.4089(2)
α(°)	90	90	90
β(º)	91.6300(7)	109.062(4)	109.0172(14)

γ(°)	90	90	90
V(Å³)	5094.45(7)	5765.7(4)	5740.21(11)
Z	4	4	4
D_x(g cm^{-3})	1.772	1.708	1.730
F(000)	2668	2928	2952
μ(mm^{-1})	6.631	3.138	3.320
Reflections:			
collected	41037	13286	110666
unique (R_{int})	9157 (0.0546)	8402 (0.0317)	12102 (0.0272)
with I>2σ(I)	8032	6490	11455
R(F) [I>2σ(I)]	0.0522	0.0418	0.0248
wR(F²) [I>2σ(I)]	0.1321	0.0848	0.0974
R(F) [all data]	0.0612	0.0646	0.0272
wR(F²) [all data]	0.1359	0.0963	0.0997
Goodness of fit	1.102	1.045	1.019
max/min Δ (e·Å^{-3})	3.82/-1.00	1.13/-1.04	2.52/-1.61
CCDC number	2062747	2062748	2062749

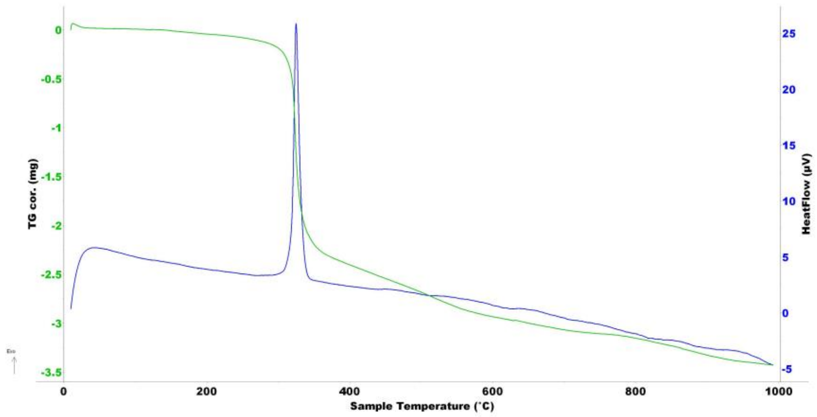

(a)

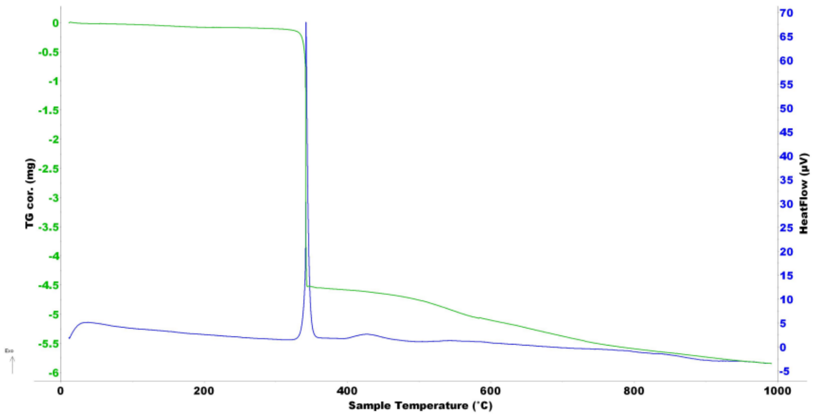

(b)

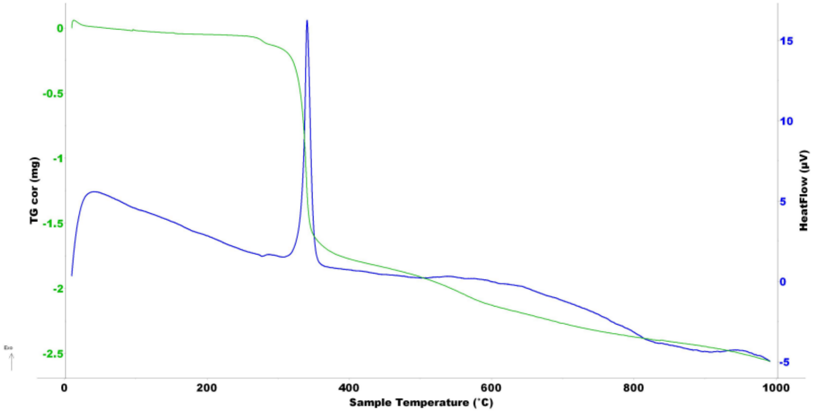

(c)

Figure S3. Thermogravimetric analysis (TGA) curves of representative of complexes (a) **2**, (b) **3**, (c) **6**.

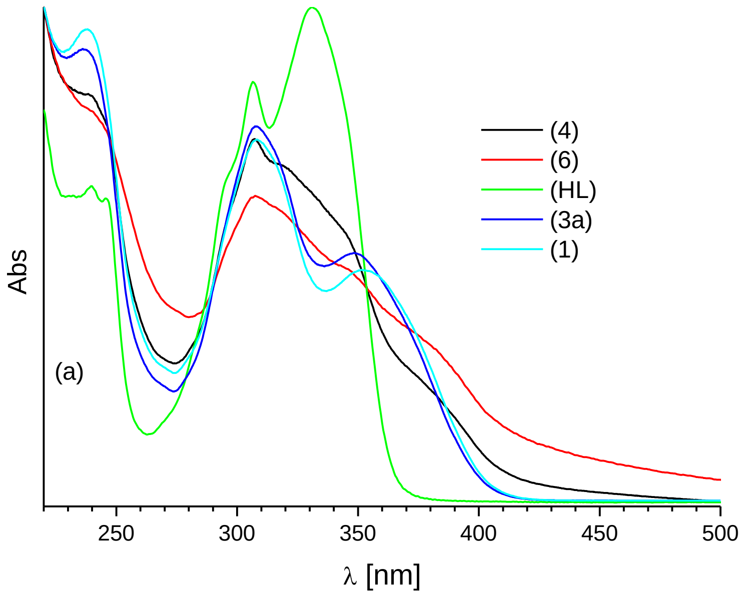

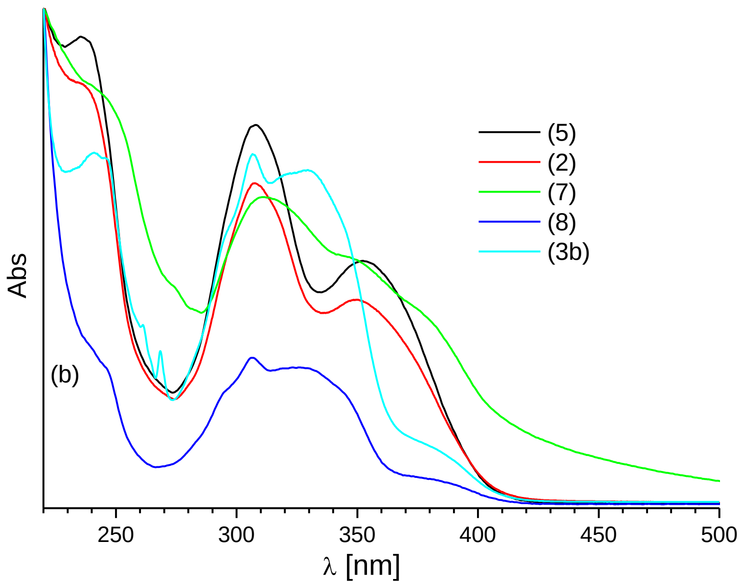

Figure S4. The UV-Vis spectra of group **A** (a) and **B** (b) compounds in acetonitrile solution.

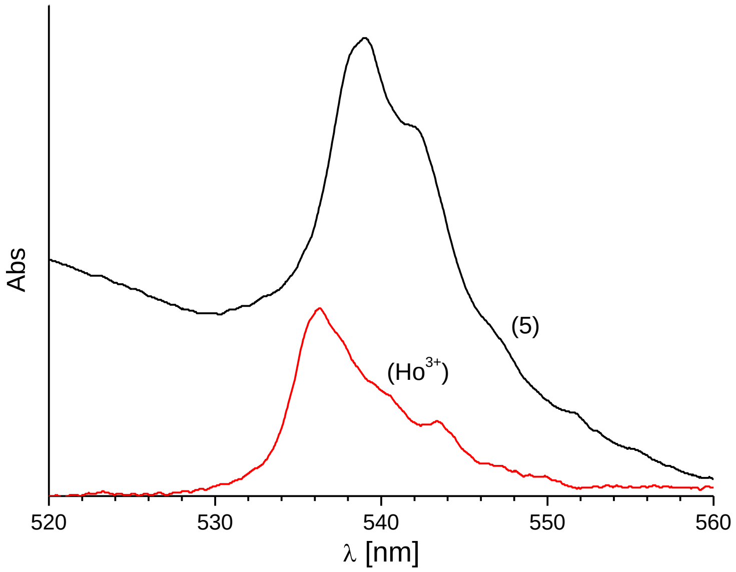

Figure S5. The absorption spectrum of Ho^{3+} ion in acetonitrile solution in $Ho(OTf)_3$ and (Ho)**5** in range $^5I_8$-$^5F_4$,$^5S_2$, c=2·× 10^{-3}.

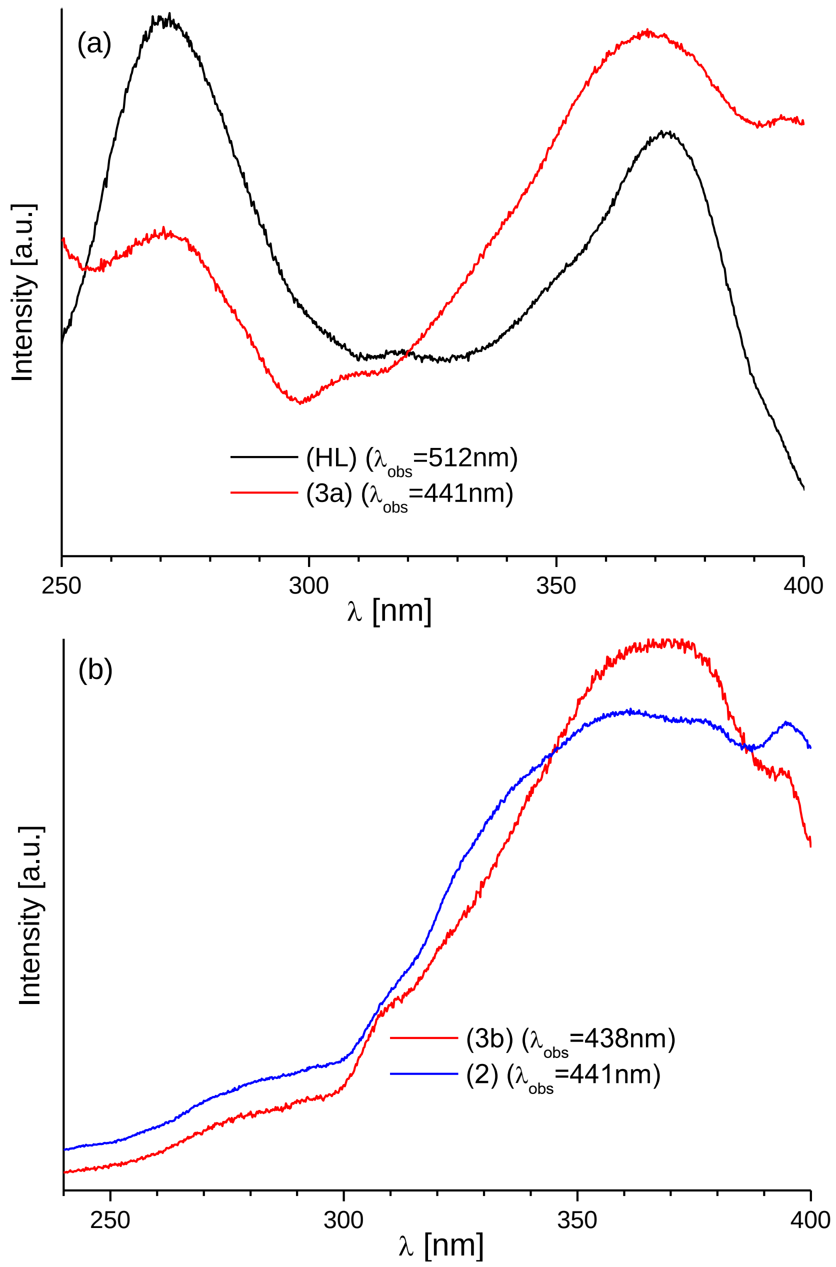

Figure S6. a–b. The excitation spectra of solid samples: (a) **HL**, **3a** and (b) **2**, **3b**.

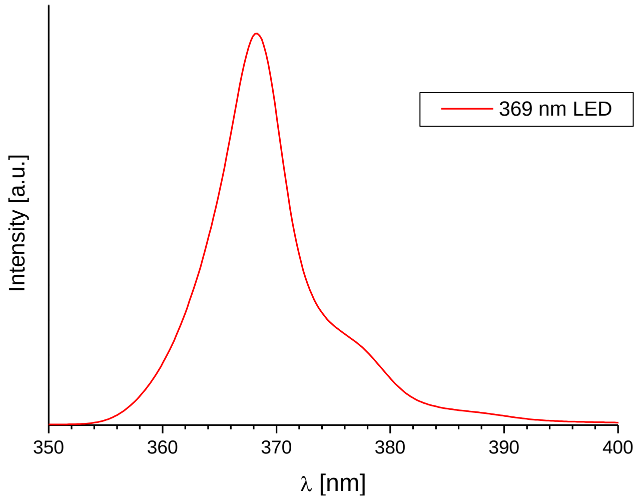

Figure S7. Spectroscopic characteristics of a 369 LED.

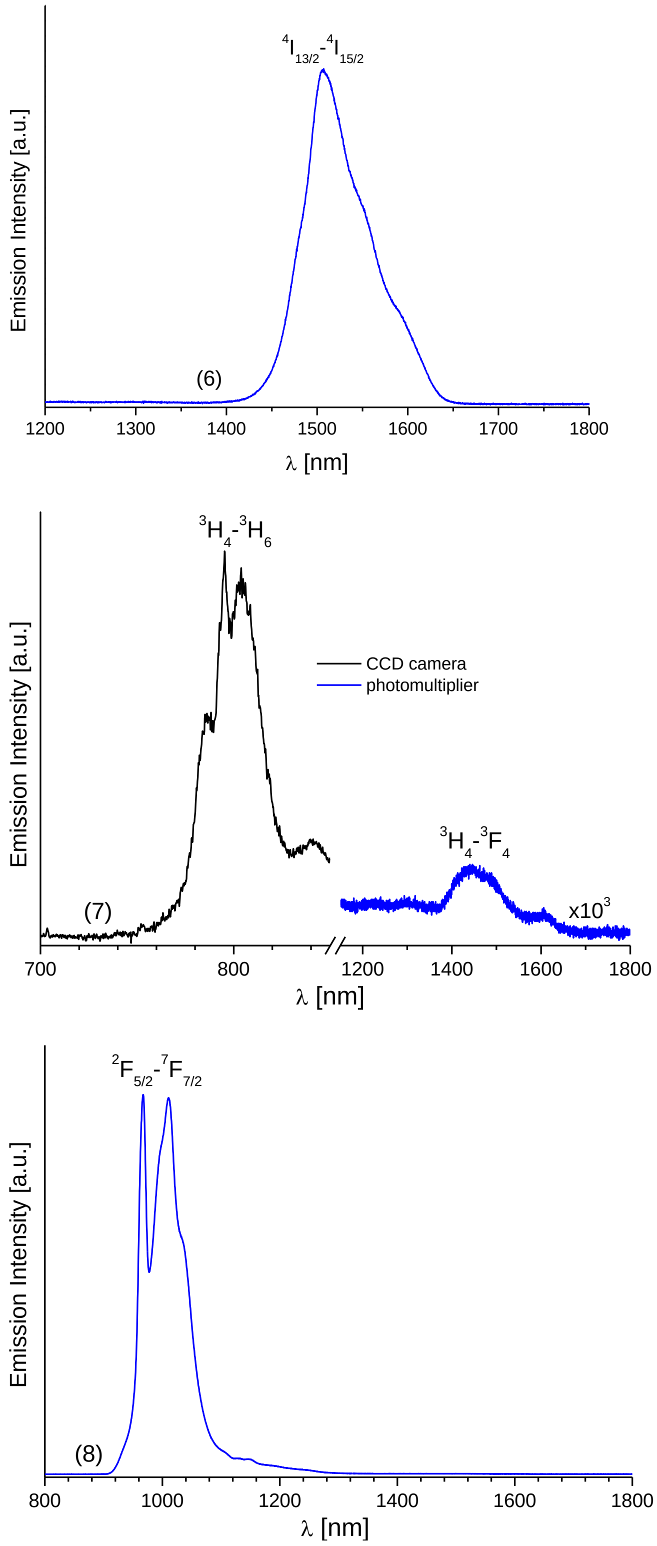

Figure S8. Photoluminescence spectra of solid samples **6**, **7** and **8** in NIR region.
